# Expression of lipid metabolism genes provides new insights into intramuscular fat deposition in Laiwu pigs

**DOI:** 10.5713/ajas.18.0225

**Published:** 2019-07-01

**Authors:** Hui Wang, Jin Wang, Dan-dan Yang, Zong-li Liu, Yong-qing Zeng, Wei Chen

**Affiliations:** 1 Shandong Provincial Key Laboratory of Animal Biotechnology and Disease Control and Prevention, College of Animal Science and Technology, Shandong Agricultural University, Tai’an, Shandong 271000, China

**Keywords:** Gene Expression, Intramuscular Fat, Laiwu Pig, Lipid Metabolism, *Longissimus dorsi* Muscle

## Abstract

**Objective:**

The objective of this study was to measure the special expression pattern of lipid metabolism genes and investigate the molecular mechanisms underlying intramuscular fat (IMF) deposition in *Longissimus dorsi* muscle of Laiwu pigs.

**Methods:**

Thirty-six pigs (Laiwu n = 18; Duroc×Landrace×Yorkshire n = 18) were used for the measurement of the backfat thickness, marbling score, IMF content, and expression of lipid metabolism genes.

**Results:**

Significant correlations were found between IMF content and the mRNA expression of lipid metabolism genes. Of the 14 fat deposition genes measured, fatty acid synthase (*FASN)* showed the strongest correlation (r = 0.75, p = 0.001) with IMF content, and of the 6 fat removal genes, carnitine palmitoyl transferase 1B (*CPT1B*) exhibited the greatest negative correlation (r = −0.66, p = 0.003) with IMF content in Laiwu pig. Multiple regression analysis showed that *CPT1B*, *FASN*, solute carrier family 27 member 1 (*SLC27A1*), and fatty acid binding protein 3 (*FABP3*) contributed 38% of the prediction value for IMF content in Laiwu pigs. Of these four variables, *CPT1B* had the greatest contribution to IMF content (14%) followed by *FASN* (11%), *SLC27A1* (9%), and *FABP3* (4%).

**Conclusion:**

Our results indicate that the combined effects of an upregulation in fat deposition genes and downregulation in fat removal genes promotes IMF deposition in Laiwu pigs.

## INTRODUCTION

Porcine intramuscular fat (IMF) and backfat thickness are essential determinants of fresh meat quality in pig production. It is widely accepted that a higher IMF content has a positive effect on the sensory experience associated with eating better quality meat [1]. The IMF content is typically higher in Chinese indigenous pig breeds than in Western pig breeds and commercial pigs. The Laiwu pig is a Chinese indigenous black pig breed that exhibits excellent meat quality with a particularly high IMF content [2,3].

The IMF is influenced by genetic and other factors, such as age, gender, and nutrition; however, genetic determinants offer the best platform for determining the molecular mechanisms of IMF deposition. Thus, genetic and genomic approaches have been used to identify quantitative trait loci and to evaluate potential candidate genes for IMF deposition in pigs [4,5]. These studies have identified many candidate genes, including some that can be used as biomarkers for the IMF trait [6,7].

The combined effects of lipid metabolism genes on IMF deposition have not yet been reported for the Laiwu pig. The objectives of this research were to measure the expression pattern of lipid metabolism genes in the *Longissimus dorsi* (LD) muscle of Laiwu pigs, and to use this information to elucidate the molecular mechanisms underlying IMF deposition in this breed.

## MATERIALS AND METHODS

This work was approved by the Institutional Animal Care and Use Ethics Committee of Shandong Agricultural University and carried out in accordance with the “Guidelines for Experimental Animals” of the Ministry of Science and Technology (Beijing, PR China).

### Animals, sample collection and lipid metabolism genes

A total of 36 castrated boars (18 Laiwu pigs and 18 Duroc× Landrace×Yorkshire [DLY] pigs) were selected and managed in two groups at a Laiwu pig breeding farm, and fed the same commercial fattening diet and water was provided *ad libitum*. The pigs were handled according to the “Regulations on Administration of Hog Slaughter” and “Good manufacturing practice for pig slaughter (GB/T 19479-2004)” of China and slaughtered following standard industry procedures. The initial body weight of the pigs was 30 kg. When the average live weight of the pigs was 80±5 kg, the pigs were slaughtered at a local commercial abattoir following standard industry procedures. Immediately after slaughter, two samples of LD muscle from the left side of the last rib of each pig were collected. One sample was frozen in liquid nitrogen and stored at −80°C for gene expression analysis, and another was stored at 4°C for analysis of IMF content.

The average backfat thickness of each carcass was measured according to the “Technical regulation for testing of carcass traits in lean-type pig (NY/T 825-2004)” of China. Briefly, the backfat thickness at the first rib, last rib and last lumbar vertebra was measured by vernier caliper, and then the average value of the three local was the average backfat thickness. Based on the “Technical regulation for determination of pork quality (NY/T 821-2004)”, marbling scores were evaluated by trained university personnel according to the National Pork Producers Council (1994), and IMF content was evaluated according to the Soxhlet petroleum-ether extraction method. IMF content is expressed as the weight percentage of wet muscle tissue.

### Lipid metabolism genes selection, RNA extraction and quantitative real-time polymerase chain reaction

Thirty lipid metabolism genes were chosen from previous studies on pigs and other animals (Table 1) for their involvement in lipogenesis, fat uptake, fatty acid esterification, lipolysis, or fatty acid oxidation. Adipocytokine and transcription factors were also considered as key molecules in the regulation of adipogenesis.

Total RNA was extracted using Trizol reagent (Invitrogen, Carlsbad, CA, USA) according to the manufacturer’s protocol. This total RNA was quantified by measuring the optical density at 260 nm, and its integrity was evaluated by 1% agarose gel electrophoresis. Ratios of absorption (260/280 nm) of all preparations were between 1.8 and 2.0. Total RNA was then reverse transcribed to cDNA using a PrimeScript RT reagent kit with gDNA Eraser (TaKaRa, Dalian, China) according to the manufacturer’s instructions.

Real-time polymerase chain reaction (PCR) was performed using SYBR Premix Ex Taq (Takara, China) and an Mx3000P Real-Time PCR System (Stratagene, La Jolla, CA, USA). Amplifications were performed in a 25 μL reaction volume containing 12.5 μL of 2× SYBR Premix ExTaq, 0.5 μL of each primer, 2 μL of diluted cDNA, 0.5 μL of ROX Reference Dye II, and sterile water. The PCR amplification was carried out as follows: 95°C for 10 s, then 40 cycles of 95°C for 5 s and 58°C for 10 s and 72°C for 15 s, followed by 1 cycle of 95°C for 1 min, 61°C for 30 s, and 95°C for 30 s to calculate the melting curve. To exclude between-run variation, all samples were amplified in triplicates and the mean was used for further analysis. Primer sets used are listed in Supplemental Table S1.

Beta-2 microglobulin, eukaryotic translation elongation factor 1 alpha 1 (*EEF1A1*), glyceraldehyde-3-phosphate dehydrogenase, peptidylprolyl isomerase A (*PPIA*), and TATA box-binding protein were amplified as endogenous control genes. The stability of the candidate reference genes was evaluated with geNorm (v3.5) [8]. The most stably expressed reference genes and their optimal number for normalization were determined. Standard curves were generated using pooled cDNA from the samples being assayed, and the ΔC*q* method was used to quantify the mRNA expression levels of lipid metabolism genes.

### Statistical analysis

The means procedure was used to calculate the mean and standard deviation values for the measured parameters (SAS Institute, v8.2, Inc., Cary, NC, USA). One-way analysis of variance followed by *t*-test were used to compare data between the two pig groups. Pearson’s correlation coefficients between carcass characteristics or lipid metabolism gene expression in LD muscle and IMF content were calculated using the CORR procedure. Principal component (PC) analysis was performed to analyze the correlations of all variables (transcription of 30 genes). Stepwise regression of SAS was used to develop equations predicting IMF content using mRNA abundance of lipid metabolism genes in LD muscle. The IMF content was the dependent variable and mRNA abundance of the 30 lipid metabolism genes was the independent variables. Data in the tables and text are presented as mean± standard error of the mean.

## RESULTS

### Backfat thickness, marbling score and intramuscular fat content

**Figure 1:**
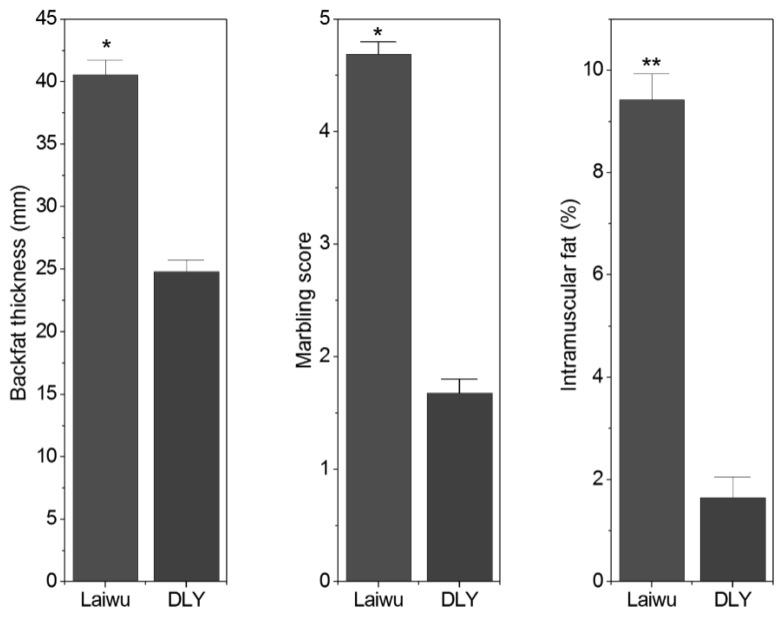
Average backfat thickness, marbling score and IMF content of Laiwu and Duroc×Landrace×Yorkshire (DLY) pigs. * p<0.05, ** p<0.01.

The average backfat thickness, marbling score and IMF content were significantly higher in Laiwu than DLY pigs (Figure 1). The IMF content was 9.43% and 1.64% for Laiwu and DLY pigs, respectively (p<0.05). Marbling score, a measure of the amount and distribution of IMF in LD muscle, was positively correlated with IMF content in Laiwu (r = 0.48, p = 0.039) and DLY pigs (r = 0.43, p = 0.046). Likewise, average backfat thickness was positively correlated with IMF content in Laiwu (r = 0.72, p = 0.008) and DLY pig (r = 0.72, p = 0.007).

### mRNA expression of lipid metabolism genes

**Figure 2:**
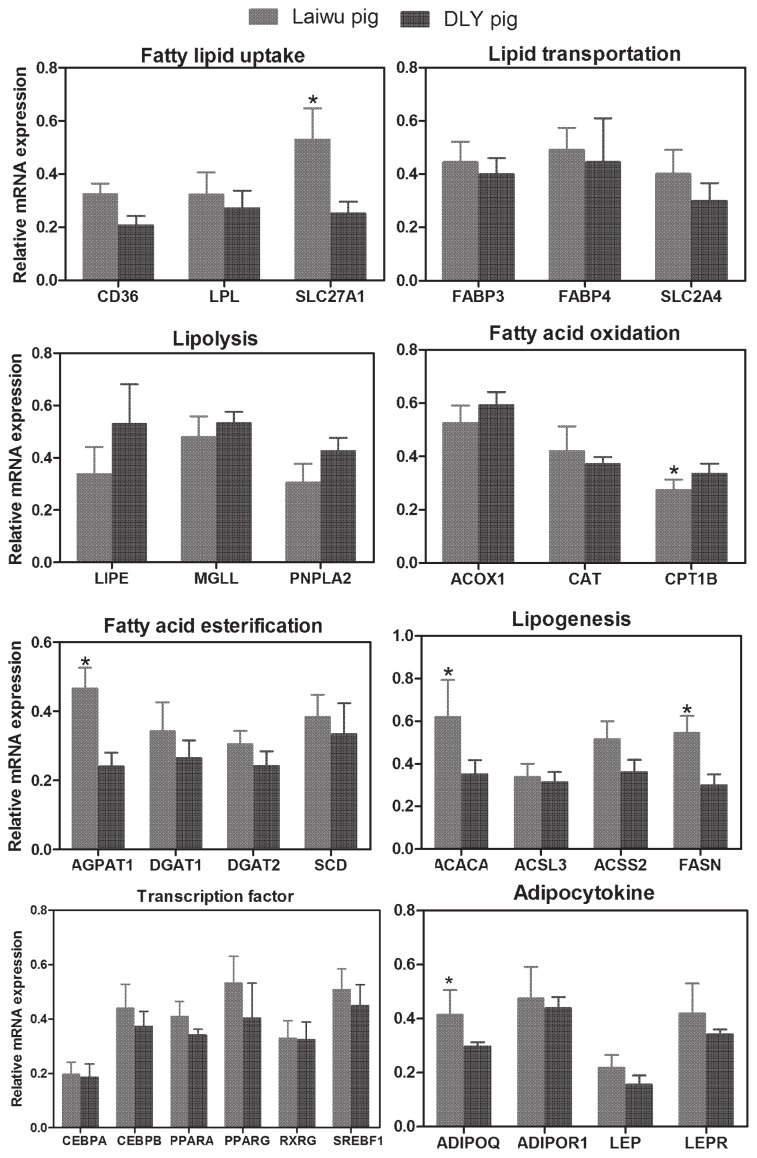
mRNA abundance of lipid metabolism genes in Laiwu and Duroc×Landrace×Yorkshire (DLY) pigs. * p<0.05.

Evaluation of candidate reference genes by geNorm showed that *EEF1A1* and *PPIA* were the most stably expressed genes. These two genes were used to normalize the expression of lipid metabolism genes. The normalized mRNA abundance of lipid metabolism genes is reported in Figure 2. The expression of acetyl-CoA carboxylase alpha (*ACACA*), fatty acid synthase (*FASN*), solute carrier family 27 member 1 (*SLC27A1*), adiponectin (*ADIPOQ*), and 1-acylglycerol-3-phosphate O-acyltransferase 1 in muscle tissue was significantly higher in Laiwu than DLY pigs. Conversely, of the fatty acid oxidation genes, only carnitine palmitoyl transferase 1B (*CPT1B*) showed a significant difference between groups, being significantly higher in DLY than Laiwu pigs (p<0.05). The expression of fatty acid binding protein 4, *FASN*, peroxisome proliferator-activated receptor gamma (*PPARG*), and *ADIPOQ* in backfat tissue was significantly higher in Laiwu than DLY pigs (p<0.05; Supplementary Figure S1). Expression of the remaining genes showed no significant differences between Laiwu and DLY pigs.

### Correlations between the expression of lipid metabolism genes and intramuscular fat content

There were many significant Pearson’s correlations between IMF content and the mRNA abundance of lipid metabolism genes (Table 2). In muscle tissue of both pig groups, IMF content was positively correlated (p<0.05) with the expression of lipid transportation and adipocytokine genes. The IMF content was also correlated (p<0.05) with mRNA abundance of diacylglycerol acyltransferase 1 (*DGAT1*), stearoyl-coenzyme A desaturase, lipase (*LIPE*), CCAAT/enhancer binding proteins (C/EBP), beta (*CEBPB*), peroxisome proliferator-activated receptor alpha (*PPARA*), *PPARG*, and sterol regulatory element binding transcription factor 1 (*SREBF1)*. Monoglyceride lipase (*MGLL*) expression was negatively correlated and *LIPE* expression positively correlated with IMF content. In Laiwu pig, IMF content was positively correlated with lipoprotein lipase, *SLC27A1*, *ACACA*, acyl-CoA synthetase short-chain family member 2 (*ACSS2*), *FASN*, and CCAAT/enhancer binding proteins (C/EBP), alpha (*CEBPA*) expression, and negatively correlated with patatin-like phospholipase domain containing 2 (*PNPLA2*), acyl-CoA oxidase 1 (*ACOX1*), and *CPT1B* expression.

However, the mRNA abundance of *ACOX1* and *PPARA* in backfat tissue was correlated with the IMF content in Laiwu pig, and catalase and *PPARA* was correlated with the IMF content in DLY pig.

### Principal component analysis and stepwise multiple regression analysis

**Figure 3:**
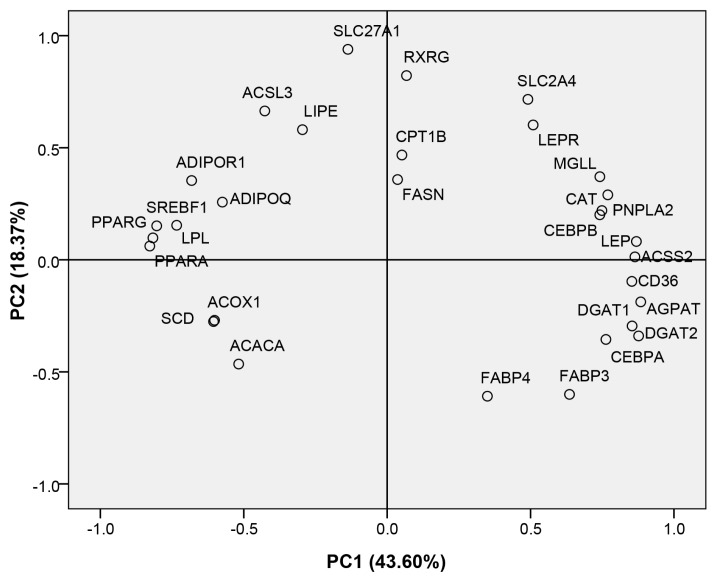
Variable scores for principal component (PC) 1 and 2 of the PC analysis. PC 1 and 2 explained 43.60% and 18.37% of the total variance, respectively. The PC plot representing variables in the rotated plan after PC analysis. The rotation method used was Varimax with Kaiser Normalization.

The PC analysis was used to determine whether any of the 30 genes analyzed significantly predicted IMF content in Laiwu pigs. The PC analysis extracted two factors, PC1 and PC2, that explained 61.97% of total data variation (43.60% and 18.37% respectively) (Figure 3). A stepwise multiple regression analysis was also performed, with four independent variables showing significant results (p<0.05, Table 3). The total prediction value for IMF content from these variables, i.e. *CPT1B*, *FASN*, *SLC27A1*, and *FABP3* expression, was 38%. Of the four variables, *CPT1B* represented the greatest contribution to IMF content (14%), followed by *FASN* (11%), *SLC27A1* (9%), and *FABP3* (4%). These results indicate that *CPT1B* may be an important factor for predicting IMF content, and thus the decrease in fatty acid oxidation caused by reduced *CPT1B* expression may be important for IMF deposition in Laiwu pigs.

## DISCUSSION

Lipogenesis is the process by which acetyl coenzyme A is converted to fatty acids [9]. Ponsuksili et al [10] reported that key genes involved in lipogenesis were upregulated in the fatter German Landrace pigs. In our study, IMF content was correlated with the expression of *ACACA*, *ACSS2*, and *FASN* in Laiwu pigs. The most significant of these was between *FASN* expression and IMF content, where the mRNA abundance of *FASN* accounted for 11% of the variability in IMF content explained by the 30 genes analyzed. Taken together, our results suggest that the upregulation in gene expression increased the capacity for fatty acid synthesis and increased the fat accumulation in skeletal muscle.

Previous research shows that SLC27A1 promotes long-chain fatty acids uptake into differentiating adipocytes [11]. In the present study, *SLC27A1* expression was correlated with IMF content in Laiwu pigs. Importantly, the mRNA abundance of *SLC27A1* accounted for 9% of the variability in IMF content explained by the 30 candidate genes we analyzed, suggesting that increased *SLC27A1* expression may promote IMF deposition in Laiwu pigs.

Diacylgycerol acyltransferase (DGAT) catalyzes the final step in triacylglycerol biosynthesis by converting diacylgycerol and fatty acyl-coenzyme A to triacylglycerol [12]. In our study, *DGAT1* and *DGAT2* expression were correlated with IMF deposition in Laiwu pigs. Therefore, *DGAT1* and *DGAT2* may play a key role in modulating fat deposition in Laiwu pigs.

Fat removal by lipolysis is an important factor in IMF de position in muscle in pigs. In this study, *LIPE* expression was positively correlated and *MGLL* and *PNPLA2* expression negatively correlated with IMF content in Laiwu pigs. In agreement with our results, a previous study reported that *MGLL* and *PNPLA2* mRNA abundance were negatively correlated with IMF content in Korean cattle steers [13]. These results suggest that decreased lipolysis may enhance fat deposition in Laiwu pigs.

Fatty acid oxidative potential may also be important for IMF deposition in skeletal muscle in pigs. A recent study reported that decreased *CPT1B* expression contributes to fat accumulation in obesity [14]. In our study, *CPT1B* expression was negatively correlated with IMF content in Laiwu pigs, with the mRNA abundance of *CPT1B* accounting for 14% of the variability in IMF content explained by the 30 genes analyzed. The *CPT1B* gene may thus be used as a genetic marker for IMF deposition in Laiwu pigs, suggesting that decreased fatty acid oxidation in muscle contributes to increasing fat deposition in this breed.

ADIPOQ can inhibit the synthesis of malonyl-coenzyme A via the cell surface receptor ADIPOR1, resulting in an increase in mitochondrial import and fatty acid oxidation [15]. We found *ADIPOQ* expression to be positively correlated with IMF content in muscle of Laiwu pigs, suggesting that *ADIPOQ* may promote lipid deposition in this breed.

*CEBPA*, *CEBPB*, *PPARA*, and *PPARG* expression were also correlated with IMF content in the Laiwu pigs of our study, with *PPARG* showing the strongest correlation with IMF content. These results indicate that an increase in *CEBPA*, *CEBPB*, *PPARA*, and *PPARG* expression leads to enhanced lipogenesis in intramuscular adipose tissue. Moreover, *SREBF1* showed a strong correlation with IMF content, suggesting that *SREBF1* may be a candidate gene for determination of lipogenic capacity. Together, these results indicate that increased expression of transcription factor genes promotes fat deposition in Laiwu pigs.

## CONCLUSION

The expression of most of the lipid metabolism genes selected for this study was significantly associated with IMF content, affirming the role of these genes in lipid deposition in muscle. Our results indicate that the combined effects of increases in fat deposition and decreases in fat removal contribute to increasing the IMF content, and that *CPT1B*, *FASN*, *SLC27A1*, and *FABP3* are predictors of IMF content in the LD muscle of Laiwu pigs.

## Supplementary Data



## Figures and Tables

**Table 1:** Information of lipid metabolism related genes

Gene name	Gene symbol	SSC	GenBank ID
Acetyl-CoA carboxylase alpha	*ACACA*	12	NM_001114269
Acyl-CoA oxidase 1	*ACOX1*	12	NM_001101028
Acyl-CoA synthetase long-chain family member 3	*ACSL3*	15	NM_001143698
Acyl-CoA synthetase short-chain family member 2	*ACSS2*	17	NM_001143695
Adiponectin	*ADIPOQ*	13	NM_214370
Adiponectin receptor 1	*ADIPOR1*	10	NM_001007193
1-acylglycerol-3-phosphate O-acyltransferase 1	*AGPAT1*	7	NM_001033008
CCAAT/enhancer binding proteins (C/EBP), alpha	*CEBPA*	6	AF103944
CCAAT/enhancer binding proteins (C/EBP), beta	*CEBPB*	17	NM_001199889
Catalase	*CAT*	2	NM_214301
CD36 molecule (thrombospondin receptor)	*CD36*	9	NM_001044622
Carnitine palmitoyl transferase 1B (muscle)	*CPT1B*	-	NM_001007191
Diacylglycerol acyltransferase 1	*DGAT1*	4	NM_214051
Diacylglycerol acyltransferase 2	*DGAT2*	9	NM_001160080
Fatty acid binding protein 3, muscle and heart	*FABP3 (H-FABP)*	6	NM_001099931
Fatty acid binding protein 4, adipocyte	*FABP4 (A-FABP)*	-	NM_001002817
Fatty acid synthase	*FASN*	12	NM_001099930
Leptin	*LEP*	18	NM_213840
Leptin receptor	*LEPR*	6	NM_001024587
Lipase, hormone-sensitive	*LIPE (HSL)*	6	NM_214315
Lipoprotein lipase	*LPL*	-	NM_214286
Monoglyceride lipase	*MGLL*	13	NM_001143718
Patatin-like phospholipase domain containing 2	*PNPLA2 (ATGL)*	2	NM_001098605
Peroxisome proliferator-activated receptor alpha	*PPARA*	5	NM_001044526
Peroxisome proliferator-activated receptor gamma	*PPARG*	13	NM_214379
Retinoid X receptor gamma	*RXRG*	4	NM_001130213
Stearoyl-coenzyme A desaturase	*SCD*	14	NM_213781
Solute carrier family 27 (fatty acid transporter), member 1	*SLC27A1 (FATP1)*	2	NM_001083931
Solute carrier family 2 (facilitated glucose transporter) member 4	*SLC2A4 (GLUT4)*	12	NM_001128433
Sterol regulatory element binding transcription factor 1	*SREBF1 (SREBP-1C)*	12	NM_214157

SSC, *Sus scrofa* chromosome.

**Table 2:** Correlation coefficients between gene expression and IMF content and backfat thickness

Genes	IMF content				Backfat thickness			
	
	Laiwu pig		DLY pig		Laiwu pig		DLY pig	
			
	r	p	r	p	r	p	r	p
Fatty lipid uptake								
*CD36*	0.47	0.051	0.21	0.404	0.27	0.275	0.36	0.067
*LPL*	**0.71**	0.039	0.39	0.051	0.39	0.071	0.27	0.058
*SLC27A1*	**0.48**	0.043	0.38	0.067	0.65	0.097	0.51	0.074
Lipid transportation								
*FABP3*	**0.39**	0.003	**0.32**	0.025	0.26	0.098	0.38	0.117
*FABP4*	**0.66**	0.000	**0.41**	0.008	0.46	0.085	0.54	0.082
*SLC2A4*	**0.39**	0.046	**0.30**	0.023	0.36	0.147	0.29	0.053
Lipogenesis								
*ACACA*	**0.43**	0.015	0.49	0.171	0.32	0.200	0.47	0.054
*ACSL3*	0.46	0.082	0.64	0.118	0.31	0.079	0.36	0.065
*ACSS2*	**0.48**	0.042	0.42	0.083	0.61	0.089	0.57	0.078
*FASN*	**0.75**	0.001	0.07	0.092	0.25	0.061	0.31	0.054
Fatty acid esterification								
*AGPAT1*	0.38	0.119	0.23	0.065	0.22	0.062	0.27	0.083
*DGAT1*	**0.51**	0.033	**0.34**	0.037	0.13	0.084	0.17	0.061
*DGAT2*	**0.37**	0.049	0.49	0.171	0.24	0.053	0.32	0.053
*SCD*	**0.48**	0.042	**0.29**	0.045	0.39	0.109	0.45	0.085
Lipolysis								
*LIPE*	**0.65**	0.003	**0.39**	0.011	0.30	0.224	0.42	0.082
*MGLL*	**−0.61**	0.009	**−0.33**	0.024	**−**0.62	0.065	**−**0.57	0.821
*PNPLA2*	**−0.58**	0.011	**−**0.29	0.065	**−**0.46	0.078	**−**0.49	0.056
Fatty acid oxidation								
*ACOX1*	**−0.56**	0.015	0.42	0.085	**−0.56**	0.016	**−**0.39	0.071
*CAT*	0.52	0.063	0.36	0.086	0.19	0.068	**0.29**	0.044
*CPT1B*	**−0.66**	0.003	**−**0.43	0.062	**−**0.49	0.087	**−**0.36	0.079
Adipocytokine								
*ADIPOQ*	**0.47**	0.047	**0.37**	0.012	0.44	0.086	0.43	0.063
*ADIPOR1*	**0.67**	0.002	**0.45**	0.019	0.57	0.082	0.39	0.104
*LEP*	**0.41**	0.044	**0.36**	0.008	0.32	0.091	0.46	0.148
*LEPR*	**0.58**	0.012	**0.29**	0.025	0.28	0.065	0.28	0.057
Transcription factors								
*CEBPA*	**0.66**	0.003	0.67	0.068	0.41	0.087	0.37	0.052
*CEBPB*	**0.74**	0.000	**0.43**	0.024	0.31	0.066	0.29	0.083
*PPARA*	**0.65**	0.003	**0.36**	0.014	**0.48**	0.029	**0.51**	0.039
*PPARG*	**0.75**	0.001	**0.29**	0.027	0.38	0.063	0.39	0.075
*RXRG*	0.39	0.112	0.32	0.065	0.21	0.218	0.59	0.175
*SREBF1*	**0.67**	0.002	**0.49**	0.038	0.39	0.065	0.41	0.052

IMF, intramuscular fat; DLY, Duroc×Landrace×Yorkshire.

**Table 3:** Stepwise multiple regression analysis for predicting IMF content using the mRNA abundance of lipid metabolism genes in Laiwu pig

Variables	Regression coefficient	SE	Partial R^2^	Pr>F
Intercept	3.19	0.21	-	<0.0001
*CPT1B*	−0.57	0.20	0.14	0.017
*FASN*	2.00	0.32	0.11	0.014
*SLC27A1*	0.37	0.11	0.09	0.006
*FABP3*	0.50	0.21	0.04	0.031
Total R^2^	-	-	0.38	-

IMF, intramuscular fat; SE, standard error; *CPT1B*, carnitine palmitoyl transferase 1B; *FASN*, fatty acid synthase; *SLC27A1*, solute carrier family 27 member 1; *FABP3*, fatty acid binding protein 3, muscle and heart.
