# Supplementary material for: Expression of lipid metabolism genes provides new insights into intramuscular fat deposition in Laiwu pigs
Source: Asian-Australas J Anim Sci. 2019 Jul 1;33(3):390–7. doi: 10.5713/ajas.18.0225 (PMC7054625; doi:10.5713/ajas.18.0225)

Supplemental Table S1. Primer sequences used for real-time PCR analysis

Gene	Forward (5'-3')	Reverse (5'-3')	Product size (bp)
Lipid metabolism related genes			
ACACA	TGGATGAGTTGGAAGTCGCT	GGAGGACGCGTAATTTCCAC	168
ACOX1	TCACAGGAAGAGCAAGGAGG	CGTGGGTGACTTGAGACTCT	242
ACSL3	GCAAGCGTGGAAAACAAACC	AGACAGACAAGCTCAGCACT	233
ACSS2	CATCCTGTGAAGGGCGAATG	CCGCCTCATGATTTTCCCTG	186
ADIPOQ	AGGCTGGATGGCGGGTAT	TGCTGAACGGTAGACATAGGC	232
ADIPOR1	AGCATCTTCCGCATCCAC	ACATCCCAAACACCACCTT	148
AGPAT1	TCTACAACGGCTGGATCCTC	TTGGAGACGACGACATAGGG	190
CEBPA	TGGACAAGAACAGCAACGA	ATCTGGAGACCCGAAACCA	317
CEBPB	TTCTACTACGAGGCGGACTG	CAGGTATGGGCTGAAGTCGA	153
CAT	AGATGGACACAGGCACATGA	CCGGATGCCATAGTCAGGAT	172
CD36	CGTGCCTATCCTCTGGCTTA	AGTTGCAGGGACACTCATGA	232
CPT1B	CCCAATTCCAGATCCGCATG	TGAGAACTTGCTGGAGACGT	155
DGAT1	CTCTTCCACTCCTGCCTGAA	ACTCGTGGAAGAAGGCTGAA	229
DGAT2	CAGTGGGTCCTGTCTTTCCT	TCGAAAGTAGCGCCAAACAG	189
FABP3	GAGTTTGATGAGACAACAGCAG	TCTTTCTCGTAAGTGCGAGTGC	188
FABP4	CTGAGATTGCCTTCAAATTG	CTTGGCTTATGCTCTCTCATA	225
FASN	CTGATCAAGGTGCTGCTGTC	CGAAGGAGTTTATGCCCACG	160
LEP	CCCTCTGAATGGTCTGGGTT	AGACCTCCCAGAGCTTTCAC	155
LEPR	ATGACTTCCTCTTGCCTGCT	TTCCCTGCAATGTTGTCTGC	196
LIPE	TGCCTTAATGTGCCTCCTGA	ATACCCGCAAAGACACCTGA	170
LPL	AAACATCCCTTTCACCCTG	TGAGTCTCTCCTGCCTTCA	195
MGLL	CGTGGATGTCATGCAGAAGG	AAGGTTGAGCACTTTCGCAG	199
PNPLA2	CGTGTACTGCAGCCTCATTC	GAACTGGATGCTGGTGTTGG	196
PPARA	ATGAACAAGGACGGGATGC	AAGAGGGAAAGGTCACTGTCG	161
PPARG	CAGCATTTCCACTCCACAC	GACACAGGCTCCACTTTGA	123
RXRG	AGTTGTGAAGGCTGCAAAGG	GCCTTTCTTCTTGCACAGCT	178
SCD	AGAAGACATCCGCCCTGAAA	TCTTGCAGGTGGGGATCAAT	164
SLC27A1	AGGAGTAGAGGGCAAAGCAG	CTGCTTCAGGTCCAGGAAGA	241
SLC2A4	CCCCTGTGTGACTCTTGGAT	CTCCCCGAAAAGTCAGTCCT	190
SREBF1	TACCGCTCCTCCATCAAT	GGTCCTTCAGAGACTTGCTT	196
Reference genes			
B2M	AAACGGAAAGCCAAATTACC	ATCCACAGCGTTAGGAGTGA	178
EEF1A1	CAGATCAGTGCTGGTTACGC	ATCGACAATGGCAGCATCAC	156
GAPDH	TCCAGTATGATTCCACCCAC	GGAGGCATTGCTGACAATCT	314
PPIA	TCTGAGCACTGGGGAGAAAG	TTTGCCATGGACAAGATGCC	192
TBP	GATGGACGTTCGGTTTAGG	AGCAGCACAGTACGAGCAA	193

Supplemental Figure S1. mRNA abundance of lipid metabolism genes in backfat tissues in Laiwu and Duroc×Landrace×Yorkshire (DLY) pigs. * $p < 0.05$.

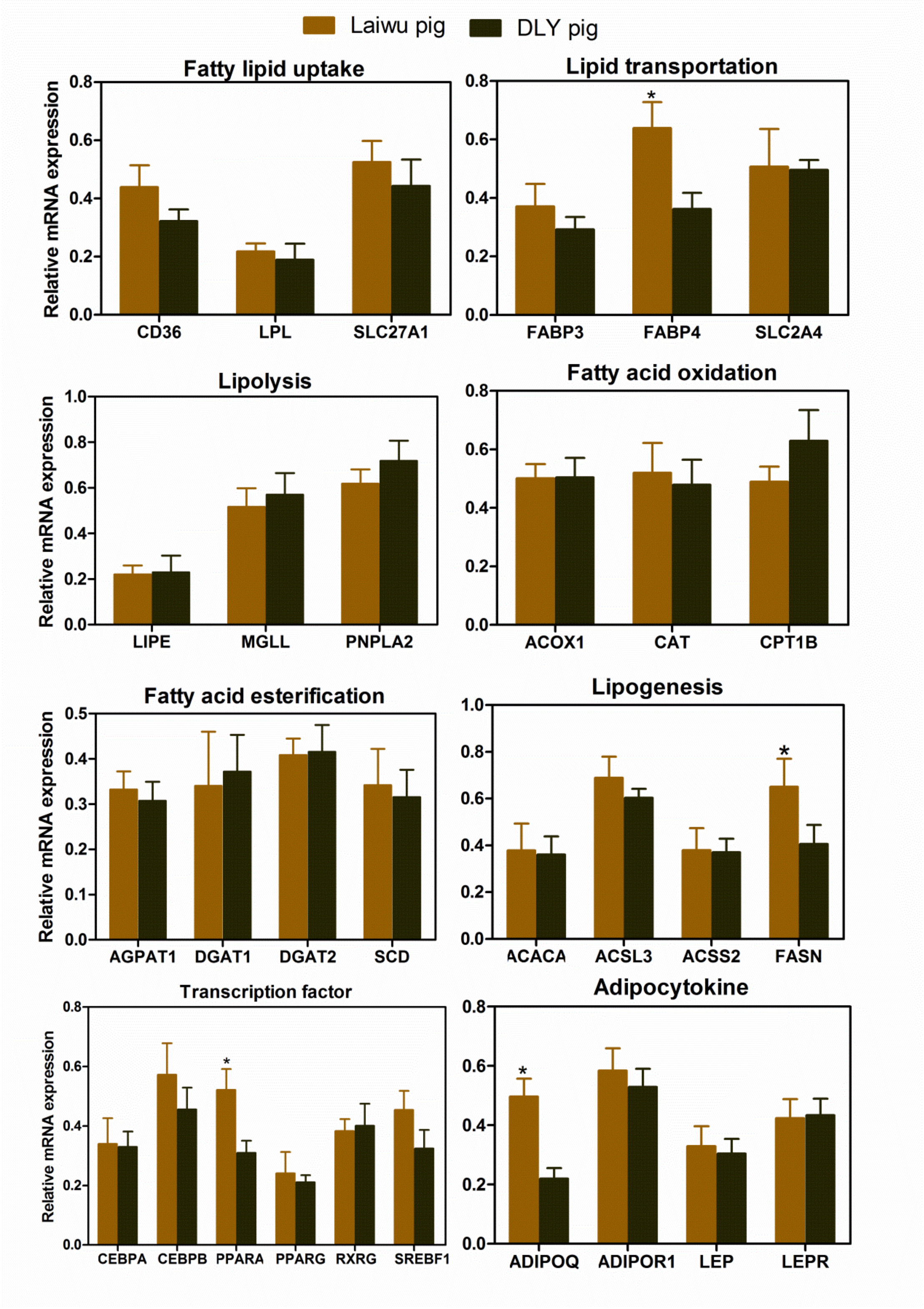

Supplement: Supplementary file 1 [file ajas-18-0225-suppl.pdf]
